# Prevalence and Predictors of Urinary Tract Infections Among Patients With Type 2 Diabetes Attending Primary Health Care Centers in Buraidah City, Saudi Arabia

**DOI:** 10.7759/cureus.86652

**Published:** 2025-06-24

**Authors:** Laila S Alrashedi, Chandra Sekhar

**Affiliations:** 1 Family Medicine, Family Medicine Academy, Qassim Health Cluster, Buraidah, SAU

**Keywords:** buraidah, glycemic control, phcc, primary heath care center (phcc), risk factors, saudi arabia, type 2 diabetes mellitus, type 2 diabetes mellitus (t2dm), type 2 diabetes patients, uti

## Abstract

Background: Urinary tract infections (UTIs) are a common occurrence among patients with diabetes mellitus (DM); it also depends on the duration and associated comorbidities. Frequent urinary tract infections (UTIs) can lead to long-term complications such as glomerulonephritis and prostatitis, and in some cases, bacteria may spread through the bloodstream, leading to sepsis. The objective of the study is to assess the prevalence and predictors of UTI among patients with type 2 DM (T2DM).

Methods: A cross-sectional study was conducted among 400 primary health care centers (PHCCs) with attendees who had T2DM. A semi-structured, self-administered questionnaire was created on Google Forms (Google Inc., Mountain View, CA) to be shared with PHCC attendees by the principal investigator through WhatsApp (Meta Platforms, Inc., Menlo Park, CA). About 72.5% (n=290) responded in our study. A convenience sampling method was used to select participants based on criteria. Data were transferred, cleaned, and analyzed with IBM SPSS Statistics software, version 21.0 (IBM Corp., Armonk, NY). Logistic regression analysis was applied to identify the predictors of UTI among patients with T2DM and their associated risk factors.

Results: This study found a significant prevalence of UTIs among patients with T2DM, with 67.9% (n=197) reporting at least one episode within 12 months. The mean age and standard deviation in the study population were 58.15 ± 12.71, with females being 54.5% (n=158). Frequent micturition (70%, n=203) and burning micturition (64.1%, n=186) were identified as major symptoms. Key risk factors included cervicitis and Foley catheter use, with notable odds ratios (OR): 3.55, CI: 1.266 to 9.959, P=0.016; OR: 2.29, CI: 0.858 to 6.160, P=0.098, respectively. Demographic factors such as age, education, and income showed no significant difference in UTI risk. The antibiotic adherence in the study group was 62.4% (n=181).

Conclusions: Nearly two-thirds (67.9%, n=197) had UTIs among patients with T2DM, with frequent and burning micturition as key symptoms. The predictors of UTI among patients with T2DM were major risk factors of cervicitis and Foley catheter use, while education and income showed no impact. The study highlights the need for early diagnosis of UTI, improved antibiotic adherence, and management strategies, including glycemic control, to reduce UTI risks.

## Introduction

Diabetes mellitus (DM) is a chronic medical condition distinguished by high blood glucose levels, which can result in the development of serious cardiovascular, ophthalmic, nephrological, or neurological disorders, as well as premature mortality [1, 2]. Type 2 DM (T2DM) is the most prevalent diabetes type, and in the past three decades, its prevalence has considerably increased [3]. The estimated prevalence of DM is 537 million adult patients worldwide as of 2021, which constitutes around 10.5% of individuals aged between 20 and 79 years globally [4, 5].

DM is a challenging health condition in the Kingdom of Saudi Arabia (KSA), with 2.5 million people affected as of 2010, which was 2.7 times higher than that in 1992 [6]. A systematic review and meta-analysis by Jarrar et al. (2023) reported a pooled prevalence of T2DM of 16.4% (95% CI: 11.6-17.5) [7]. KSA is one of the top five countries with the highest age-adjusted diabetes prevalence [4]. The mortality from DM is the second highest in the Middle East and Northern Africa region (20.2%) [4]. Meanwhile, KSA has the highest mortality related to T2DM among the Gulf Cooperation Council (GCC) countries [8].

Diabetic patients are immunocompromised because of uncontrolled hyperglycemia, hence contributing to the development of various infections, such as skin, soft tissue, urinary, respiratory tract, or surgical site infections [9]. Rates of hospitalization due to infection are estimated to be 2.6 to 15.7 times (depending on the infection type) higher among patients with diabetes than those without DM [10].

Furthermore, bladder dysfunction due to nephropathy and glucosuria can contribute to the emergence of urinary tract infections (UTIs) [11]. The pooled prevalence of UTIs was estimated to be 11.5% (95% CI: 7.8-16.7%) among patients with T2DM in the systematic review and meta-analysis by Salari et al. (2022) [11], and an even higher rate (25.3%) was reported in a cross-sectional study among Saudi patients with diabetes [12]. Female sex, body mass index >30 kg/m², insulin therapy, hypertension, and nephropathy are associated with an increased risk of developing UTI among patients with DM [13].

An Ethiopian study [14] stated that UTI prevalence was 22.3% among patients with diabetes and concluded that regular screening is recommended for early UTI detection. Also, a study from Germany [15] revealed that prompt UTI diagnosis and appropriate antibiotic use are crucial. A study from India [16] mentioned caution about risk factors for early diagnosis of UTI. An study from the USA [17] stated that patients with diabetes have a 25% higher chance of UTI than those without, and another study stated that it is significantly associated with female gender, illiteracy, and prior UTIs [18]. A Sudanese study [19] stated that 19.5% of UTI prevalence was among patients with diabetes without notable risk factors. A Malaysian study [20] reported a UTI prevalence of 40.2%, while an Egyptian study [21] found a prevalence of 51.3% among patients with diabetes.

DM is a chronic medical condition that poses significant health risks and can lead to various complications, including UTIs. UTIs among patients with diabetes can affect their health and overall quality of life. It is crucial to gain a comprehensive understanding of the prevalence and predictors of UTIs in this population in order to develop effective prevention and management strategies. By focusing on this topic in a local context, the study will provide unique insights into the prevalence and predictors of UTIs among patients with diabetes in Saudi Arabia.

The findings of this study provide some practical implications for healthcare providers, policymakers, and researchers. By identifying the specific risk factors associated with UTIs in patients with T2DM, targeted interventions can be developed to prevent and manage UTIs more effectively. This, in turn, will improve the overall care and well-being of patients with diabetes in the region.

This study aimed to determine the prevalence, predictors, risk factors, and their associations with UTIs among patients with T2DM at primary health care centers (PHCCs) of Buraidah, Saudi Arabia.

## Materials and methods

Study design and population

This cross-sectional study was conducted among patients with T2DM attending PHCCs in Buraidah, Saudi Arabia. The inclusion criteria were patients with T2DM residing in Buraidah who were 30 years or older. The exclusion criteria included patients with type 1 DM (T1DM), gestational diabetes, and non-Arabic-speaking patients.

Sampling and sample size

The sample size for this study was calculated using the online web-based sample size calculator OpenEpi (Sullivan, Dean, & Soe, OpenEpi: Open source epidemiologic statistics for public health (Version 3.01, April 6, 2013) (Software), Retrieved from http://www.OpenEpi.com) [22]. The population of Buraidah is 677,647, as per the General Authority of Statistics 2022 [23]. The prevalence of UTI noted among the population with diabetes was anticipated to be 50%, with confidence limits of 95%, and the design effect was 1.0. Based on the above parameters, the sample estimate was 384.

Sampling method 

As per the Qassim Health Cluster (QHC) information, 43 PHCCs are functional [24] in Buraidah. Of these, 10 PHCCs were selected based on geographic location (two PHCCs from the north side, two from the south, two from the east side, two from the west side, and lastly two PHCCs from central Buraidah). For each selected PHCC, the participant recruitment was done using a convenience sampling method.

Data collection tool and procedure

Once we formed our research idea, we searched the literature and found some studies from Saudi Arabia [25], India [16], and the USA [17]. Then, we developed a questionnaire based on our study objectives. Once the questionnaire was constructed for the validation process, it was reviewed with colleagues, research experience staff, and family medicine consultants. After the initial review, the questionnaire was subjected to back-to-back translation to improve its validity.

The questionnaire consisted of two sections. The first section denoted demographic characteristics such as age, gender, marital status, education, occupation, income, residence status, and anthropometric measurements to find the body mass index. The second section comprised prevalence and risk factors like UTI among diabetes disease-related factors, glycemic control factors, lifestyle factors of individuals, comorbidity, personal and family history factors, etc., which were included to find the risk factors association and predictors of UTI among patients with diabetes (Appendix A). Then, we shared the semi-structured, self-administered questionnaire through WhatsApp (Meta Platforms Inc., Menlo Park, CA) to selected PHCC attendees, based on our criteria.

Pilot study 

A pilot study was conducted in the field to determine technical feasibility and ensure a good presentation and order of the questions among 20 patients with T2DM. The pilot study sample was not included in the main study sample. After the pilot study, our questionnaire did not change.

Ethical considerations

The data collection process was started after obtaining the Qassim Regional Ethics Committee's approval, Buraidah (approval number: 607-45-15281, dated May 22, 2024). Before recruiting the sample, the permission of the concerned PHCC directors was obtained. Each participant gave informed consent. Informed consent was inferred as they agreed, and consent was displayed on the first page of the Google Forms (Google Inc., Mountain View, CA). Individuals' information was maintained confidentially, and data were shared with any private or governmental agencies.

Statistical analysis

For the demographic characteristics, frequency and percentages were calculated. Means and standard deviations were calculated for the continuous variables in the study. For all the categorical variables, the chi-square test was applied. The level of statistical significance was taken as the probability (P) value being less than or equal to 0.05. An independent t-test was applied to compare the mean age of the study population with the prevalence of UTI among the population with diabetes. All the statistical tests were analyzed using IBM SPSS Statistics software, version 21 (IBM Corp., Armonk, NY). 

## Results

We distributed the questionnaire to approximately 400 participants. The response rate in the study population was 72.5% (290/400). The study population's mean age and SD were 58.15 ± 12.71 years.

Table 1 shows that among the 290 respondents, 54.5% (n = 158) were female and 45.5% (n = 132) were male. The majority of participants were aged 60 years or older (42.8%, n = 124), followed by those aged between 46 and 60 years (40.3%, n = 124). Most participants were married (75.5%, n = 219), and a significant proportion held a bachelor’s degree (33.1%, n = 96), followed by those with a diploma (16.9%, n = 49) and a postgraduate degree (14.1%, n = 41). In terms of employment, 31.7% (n = 92) were government employees, 31.0% (n = 90) were unemployed, and 23.1% (n = 67) were retired. Monthly income levels varied, with 30.7% (n = 89) earning between 10,001 and 15,000 Saudi Riyals (SR), 20.0% (n = 58) earning 5,001-10,000 SR, and 17.9% (n = 52) earning over 20,000 SR (Table 1).

**Table 1:** Demographic characteristics of the patients with T2DM attending the PHCCs of Buraidah (n=290) N: number of participants; %: percentage; yrs: years; SR: Saudi Riyal; T2DM: type 2 diabetes mellitus; PHCCs: primary health care centers

Demographic factors	Number of participants (N)	Percentage (%)
Age ± SD	58.15 ± 12.71	
Age category		
31-45 yrs	49	16.9
46-60 yrs	117	40.3
>60 yrs	124	42.8
Gender		
Male	132	45.5
Female	158	54.5
Marital status		
Single	14	4.8
Married	219	75.5
Divorced	22	7.6
Widowed	35	12.1
Education		
No formal education	23	7.9
Less than high school	45	15.5
Secondary school	36	12.4
Diploma	49	16.9
Bachelor degree	96	33.1
Postgraduation degree	41	14.1
Occupation		
Government Employee	92	31.7
Private employee	41	14.1
Retired	67	23.1
Unemployed	90	31.0
Income		
< 5,000 SR/month	38	13.1
5,001-10,000 SR/month	58	20.0
10,001-15,000 SR/month	89	30.7
15,001-20,000 SR/month	53	18.3
>20,000 SR/month	52	17.9

Table 2 represents diabetes management strategies, with 86.6% (n = 251) of participants on oral hypoglycemic agents, 44.5% (n = 129) using insulin alone, and 42.4% (n = 123) using a combination of pills and insulin. Insulin pump use was reported by 12.4% (n = 36), and 28.3% (n = 82) were receiving injections. Physical activity was reported by only 34.8% (n = 101) of participants, while 21.0% (n = 61) were smokers. A majority (75.5%, n = 219) had a family history of diabetes (Table 2). 

**Table 2:** Nonpharmacological and pharmacological management of the population with T2DM N: number of participants; %: percentage; T2DM: type 2 diabetes mellitus

Variables	N (%)	Yes (%)	Total (%)
Smoking	229 (79.0)	61 (21)	290 (100%)
Physical activity	189 (65.2)	101 (34.8)	290 (100%)
Family history	71 (24.5)	219 (75.5)	290 (100%)
Treatment with pills	39 (13.4)	251 (86.6)	290 (100%)
Insulin alone	161 (55.5)	129 (44.5)	290 (100%)
Pills + insulin	167 (57.6)	123 (42.4)	290 (100%)
Insulin pumps	254 (87.6)	36 (12.4)	290 (100%)
Injections	208 (71.7)	82 (28.3)	290 (100%)

Table 3 shows diabetes control based on the participant self-rating variable; 43.1% (n = 125) of participants reported good DM control, whereas 9% (n = 26) had poor DM control, and 47.9% (n = 139) had medium DM control. 

**Table 3:** Self-reported responses of study participants regarding diabetes control DM: diabetes mellitus; N: number of participants; %: percentage

DM control	Number of participants (n)	Percentage (%)
Good control	125	43.1
Medium control	139	47.9
Poor control	26	9.0
Total	290	100.0

**Figure 1:**
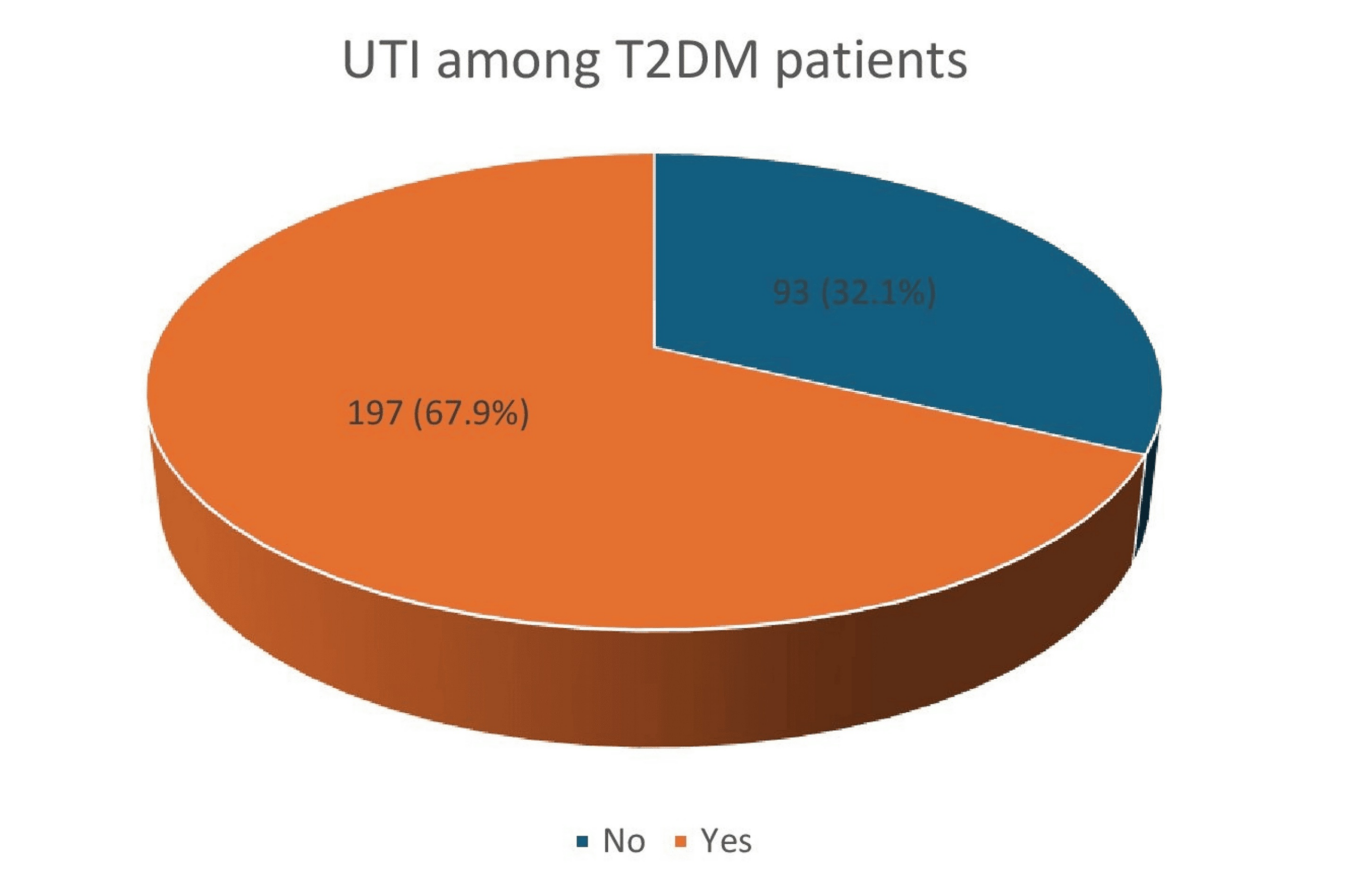
Prevalence of UTI among patients with T2DM in the study population UTI: urinary tract infection; T2DM: type 2 diabetes mellitus

Table 4 shows that comorbidities were prevalent among the study population. Hypertension was present in 39.3% (n = 114) of participants, and 35.9% (n = 104) had lipid disorders. Chronic diseases were reported by 51.0% (n = 148) of the participants overall. Coronary artery disease (16.2%, n = 47), kidney disease (7.2%, n = 21), retinopathy (12.4%, n = 36), and neuropathy (3.1%, n = 9) were less frequently reported (Table 4). Figure 1 stated that about 67.9% (n=197) of patients with T2DM had a UTI within a duration of 12 months. 

**Table 4:** Comorbidity status of the study population from Buraidah (n=290) N: number of participants; %: percentage

Comorbidity cariables	N (%)	Yes (%)	Total (%)
Chronic disease	142 (49.0)	148 (51.0)	290 (100%)
Hypertension	100 (34.5)	114 (39.3)	290 (100%)
Lipid disorders	98 (33.8)	104 (35.9)	290 (100%)
Coronary artery disease (CAD)	142 (49.0)	47 (16.2)	290 (100%)
Kidney disease	160 (55.2)	21 (7.2)	290 (100%)
Retinopathy	155 (53.4)	36 (12.4)	290 (100%)
Neuropathy	170 (58.6)	9 (3.1)	290 (100%)
Any other	158 (54.5)	34 (11.7)	290 (100%)

Table 5 presents the most common symptoms of UTI, including frequent urination (70.0%, n = 203) and burning urination (64.1%, n = 186). Abdominal pain and loin pain were reported by 46.6% (n = 135) and 34.5% (n = 100), respectively. Fever was reported in 20.3% (n = 59), while high fever (11.7%, n = 34) and nausea/vomiting (18.6%, n = 54) were less common. Only 9.3% (n = 27) of participants reported complications following UTIs (Table 5). In the present study, common risk factors identified among patients included Foley’s catheter insertion (19.0%, n = 55), renal calculi (11.4%, n = 53), benign prostatic hyperplasia (18.3%, n = 53), and cervicitis (18.3%, n = 53). About 62.4% (n = 181) of those treated for UTIs reported adherence to prescribed antibiotic regimens (Table 5).

**Table 5:** UTI symptoms, risk factors, and complications among patients with T2DM in the study group N: number of participants; %: percentage; UTI: urinary tract infection; T2DM: type 2 diabetes mellitus

Variables	N (%)	Yes (%)	Total
Symptoms of UTI among patients with T2DM			
Burning micturition	104 (35.9)	186 (64.1)	290 (100%)
Frequent micturition	87 (30.0)	203 (70.0)	290 (100%)
Abdominal pain	155 (53.4)	135 (46.6)	290 (100%)
Loin pain	190 65.5)	100 (34.5)	290 (100%)
Fever	231 (79.7)	59 (20.3)	290 (100%)
High fever	256 (88.3)	34 (11.7)	290 (100%)
Nausea and vomiting	233 (80.3)	54 (18.6)	290 (100%)
Complications following UTI	263 (90.7)	27 (9.3)	290 (100%)
Risk factors of UTI			
Foley’s catheter insertion	123 (42.4)	55 (19.0)	178 (100%)
Renal calculi	139 (47.9)	33 (11.4)	172 (100%)
Stricture urethra	148 (51.0)	23 (7.9)	171 (100%)
Benign prostatic hyperplasia	136 (46.9)	53 (18.3)	189 (100%)
Cervicitis	128 (44.1)	53 (18.3)	181 (100%)
UTI antibiotic adherence	61 (21.0)	181 (62.4)	242 (100%)

Table 6 presents a high prevalence of UTIs among patients with T2DM within 12 months. Specifically, 35.9% (n = 104) reported one to two episodes, 27.9% (n = 81) had three to four episodes, 5.5% (n = 16) experienced more than five episodes, and 32.1% (n = 93) of the study population did not have any UTI. 

**Table 6:** Frequency of UTI among patients with T2DM in the study population N: number of participants; %: percentage; UTI: urinary tract infection; T2DM: type 2 diabetes mellitus

Number of UTI occurences within 12 months	N (n)	Percentage (%)
1-2 times	104	35.9
3-4 times	81	27.9
More than 5 times	16	5.5
No UTI	89	30.7
Total	290	100

Table 7 states that the mean age and SD for the UTI among patients with T2DM were 59.04 ± 12.47 years, and the mean age and SD among the non-UTI patients with T2DM were 56.28 ± 13.07 years. The UTI prevalence among females was 70.3% (n = 111), whereas it was 65.2% (n = 86) among males. When looking at education, the results showed no significant association between education level and the chance of getting a UTI among T2DM patients. People with less than a high school education had the highest UTI rate (77.8%, n = 35), followed by those with secondary school (72.2%, n = 26) and diploma holders (67.3%, n = 33). However, people with higher education, like a bachelor’s or postgraduate degree, had lower UTI rates (64.6%, n = 62, and 63.4%, n = 26). Even though there were some differences in the prevalence of UTI at different levels of education, they were not statistically significant (P>0.05). As for monthly income, the highest UTI rate was among those earning between 10,001 and 15,000 SR/month (74.2%, n = 66), followed by those earning 5,001-10,000 SR/month (70.7%, n = 41). In contrast, people earning more than 20,000 SR/month had a lower UTI rate (57.7%, n = 30), and those with income below 5,000 SR/month had 63.2% (n = 24). Again, the differences were not statistically significant, which means income does not seem to have a substantial effect on UTI risk in this group in our study. In relation to glycemic control among patients with T2DM with glycated hemoglobin (HbA1c) less than 7%, the prevalence of UTI was 63.8% (n = 60), whereas among patients with T2DM with > 7% HbA1C, the prevalence was 69.9% (n = 137) (odds ratio (OR) = 1.31, CI: 0.782 to 2.213) (Table 7).

**Table 7:** Sociodemographic factors and glycaemic control status association with UTI among T2DM patients in the study population. N: number of participants; %: percentage; SR: Saudi Riyal; UTI: urinary tract infection; T2DM: type 2 diabetes mellitus; T: independent t-test; X2: chi-square test; P: probability; OR: odds ratio

Sociodemographic variables	No UTI among patients with T2DM	UTI among patients with T2DM	P-value
Age	56.28 ± 13.07; N=93	59.04 ± 12.47; N=197	T=-1.732, P=0.084
Gender			
Male	46 (34.8%)	86 (65.2%)	X^2^ = 0.859, P = 0.354,OR=1.26
Female	47 (29.7%)	111 (70.3%)	
Education			
No formal education	8 (34.8%)	15 (65.2%)	X^2^ = 3.270, P = 0.658
Less than high school	10 (22.2%)	35 (77.8%)	
Secondary school	10 (27.8%)	26 (72.2%)	
Diploma	16 (32.7%)	33 (67.3%)	
Bachelor degree	34 (35.4%)	62 (64.6%)	
Postgraduation degree	15 (36.6%)	26 (63.4%)	
Monthly income			
Income < 5,000 SR/month	14 (36.8%)	24 (63.2%)	X^2^ – 4.686, P = 0.321
5,001-10,000 SR/month	17 (29.3%)	41 (70.7%)	
10,001-15,000 SR/month	23 (25.8%)	66 (74.2%)	
15,001-20,000 SR/month	17 (32.1%)	36 (67.9%)	
>20,000 SR/month	22 (42.3%)	30 (57.7%)	
Glycemic control (HbA1C)			
< or = 7%	34 (36.2%)	60 (63.8%)	X^2^ – 1.074, P = 0.300,OR=1.31
>7%	59 (30.1%)	137(69.9%)	

Table 8 represents the regression analysis of the risk factors for UTI among patients with T2DM. Of all the risk factors studied in our study, patients with a cervicitis risk factor were 3.55 times more likely to develop a UTI (P = 0.016), followed by those who underwent Foley catheter insertion, who were 2.29 times more likely to develop a UTI (P = 0.098).

**Table 8:** Regression analysis of predictors of risk factors of UTI among the study population with T2DM OR: odds ratio; AOR: adjusted odds ratio; Ref: reference category; UTI: urinary tract infection; T2DM: type 2 diabetes mellitus

Variables	Categories	OR	AOR	P-value	Confidence interval
Smoking	No/Yes (Ref)	0.797	1.522	0.367	0.611 to 3.790
Physical activity	No/Yes (Ref)	0.636	.683	0.285	0.339 to 1.374
Risk factor: Foley catheter	No/Yes (Ref)	2.474	2.299	0.098	0.858 to 6.160
Risk factor: renal calculi	No/Yes (Ref)	1.648	1.192	0.732	0.436 to 3.259
Risk factor: urethral strictures	No/Yes (Ref)	0.747	.289	0.056	0.081 to 1.032
Risk factor: benign prostatic hyperplasia	No/Yes (Ref)	1.221	1.376	0.512	0.531 to 3.565
Risk factor: cervicitis	No/Yes (Ref)	2.368	3.550	0.016	1.266 to 9.959

## Discussion

This study highlights a notably high prevalence of UTIs among patients with T2DM, with over two-thirds (67.9%) experiencing at least one episode in the previous year. The high prevalence of UTI in our study could be due to the elderly population, uncontrolled blood sugar, non-compliance with medication, and also the global high prevalence of T2DM, including in Saudi Arabia. This study's prevalence of UTI aligns with some existing literature and states that some patients with T2DM are more prone to infections, impaired immune response, and associated comorbidities [12]. 

Another study was conducted among 1000 patients with T1DM and T2DM, and the prevalence of UTI was 25.3% [12]. This study stated that patients with diabetes are more prone to infections and that there is a poor immune response. This low prevalence could be due to a study conducted more than 12 years ago; at that time, the global prevalence of diabetes could have been lower. UTI prevalence among patients with diabetes from a study done in Kuwait was reported as 35% [26]. In a study done in Malaysia, the prevalence of UTI among patients with diabetes was 40.2% [20]; in Egypt, it was 51.3% among patients with diabetes [19], and a study in Nepal reported a prevalence of UTI among patients with diabetes as 54.7% [27]. In another study from Jazan, among 440 study participants with T2DM, the UTI prevalence was 60.9% [25]. The different prevalence of UTI among patients with diabetes depends on geographical variations, study settings, the year of the study, the age group involved in the studies, and associated comorbidities.

The current study population had a mean age of 58.15 ± 12.71 years. Another study revealed that the mean age and SD were 44.36 ± 14.81 years [25]. An Indian study revealed that the mean age of the study population was 52.18 ± 9.06 years [16]. In the present study, the prevalence of UTI among females was 70.3%, and among males was 65.2%. Many studies have reported that female sex is significantly associated with UTI among T2DM patients. Notably, a study from the USA reported a 25% higher risk of UTI among diabetics [17]. In studies from Ethiopia [18], Egypt [21], and Romania [28], it was shown to be 3.47 times more common among females (P<0.001). A possible reason could be that the length of the urethra among females is shorter compared to males.

In the current study, chronic disease prevalence (which included hypertension, lipid disorder, cardiovascular diseases, and others) was reported as 51%. In another study, an almost similar percentage of chronic disease comorbidity was 47.6% [25], and a low prevalence of chronic disease of 24.5% was reported in a study (OR: 4.87, 95% CI: 2.76 to 8.59) [14].

In our current study, the mean HbA1C level and SD were 8.40 ± 1.32, which was a bit higher and could be due to more elderly patients with diabetes who had a longer duration of diabetes. In the present study, those patients who had HbA1C levels >7% had 1.31 times more chances of getting a UTI, and a median HbA1C level of 8% was observed in the study from Romania [28]. This indicates that stringent glycemic control is required. Similar results were observed in other studies [28, 29]. This higher glycemic status could be due to a longer duration of T2DM. Strict lifestyle modifications and good adherence to antidiabetic medication play a role in the somewhat controlled HbA1C level.

In the current study, 35.9% reported one to two episodes, 27.9% had three to four episodes, and 5.5% experienced more than five episodes. A study from Jazan reported that 60.6% of patients had one to two UTIs a year, 25.6% had three to four UTIs a year, and 13.8% had > five UTIs a year. This high prevalence could be due to the endocrinology hospital setting [25]. The most common UTI symptoms included frequent micturition (70.0%) and burning micturition (64.1%). Our study reported abdominal and loin pain in 46.6% and 34.5%, respectively. The greater the number of symptom responses presented in a single patient. Hence, the prevalence of UTI symptoms is shown to be a bit high. A study from Jazan about symptoms of burning micturition was 26.6%, frequent urination was 19.1%, and abdominal pain was 22% among the diabetic patients [25].

In the current study, logistic regression analysis revealed that patients with a cervicitis risk factor were 3.55 times more likely to develop a UTI than those without cervicitis, while those with Foley catheter insertion had a 2.29 times higher risk of developing a UTI. Many studies from different countries, including CDC guidelines, stated that cervicitis is one of the important factors in sexually transmitted diseases, including UTIs, and the risk increases manyfold [30-33]. Higher UTI rates were associated with less than high school education and specific income brackets (10,000 to 15,000 SR/month), though differences were not statistically significant (P > 0.05).

The key strengths in the study are a large sample of patients with T2DM from multiple PHCCs and one of the common iceberg disease explorations. Some of the limitations observed in the study are that self-administered questionnaires may have some misunderstanding of the questions; additionally, the majority of participants were elderly, which made it more challenging for them to use social media to respond to the questionnaire. And the study design is a cross-sectional study, which can't establish causal inferences/associations. Another factor is that the study was designed only in Buraidah city due to limitations of time in the family medicine residency program. The results of the study may not generalize to the entire population of Qassim. However, these results are helpful for policymakers in the implementation of adequate control of UTI among patients with diabetes attending PHCCs.

## Conclusions

Based on the study results, UTI prevalence among patients with T2DM was high, and awareness of UTI risk factors and diabetes control is needed. There is a need to address the behavior modification of patients, and UTI antibiotic adherence needs to improve. Reinforcement of patient education and early identification of risk factors are essential for the reduction of healthcare costs. Diabetes control and adherence to antibiotic treatment are the key steps in the reduction of the problem. Future long-term multicentric studies in large geographical areas (multiple provinces) are required to substantiate the generalizability of the study results at the country level.

## Appendices

Appendix A

Study Questionnaire

Age: -------------(Completed years)

Gender: 1. Male          2. Female        Height: ------Cms         Weight: ------- Kgs

Marital Status: 1. Single         2. Married        3. Divorced     4. Widowed

Education:    1. No formal education  2. Less than high school 3. Secondary school          4. Diploma 5. Bachelor’s degree 6. Post-graduation and 

Occupation: 1. Government employee          2. Private employee    3. Retired        4. Unemployed

Income/month: 1. Less than 5000 SR  2. 5001-10000 SR  3. 10001-15000 SR 4. 15001-20000 SR 5. > 20,000 SR 

Smoking: 1. No 2. Yes            Physical activity: 1. No 2. Yes.

Do you have a close relative diagnosed with DM? 1. No 2. Yes

Duration of diabetes: ------------------Years

How do you rate your diabetes control: 1. well controlled, 2. moderately controlled, 3. poorly controlled 

Medications used: 1. Diet and exercise 2.  Insulin 3. Pills 4. Pills and Insulins 5. Insulin pump 6. Injections 

Treatment adherence to Diabetes medication (6 days out of seven days consumed/ course received as per the doctor's advice - self-reported statement of participant): 1. Adherent 2. Non-adherent

Co-morbidities: 1. No 2. Yes

If yes, 1. HTN 2. Lipid disorders 3. CAD 4. Kidney disease 5. Retinopathy 6. Neuropathy 7. Any other (Specify)----------

Previous history of UTI in the last 12 months: 1. No 2. Yes

How often have you had a urinary tract infection in the previous 12 months?  1. None 2. 1-2 times  3. 3-4 times  4. 5 or more times

Symptoms of urinary tract infections: 1. Burning micturition 2. Frequent micturition 3. Abdominal pain 4. Loin pain 5. Fever 6. High fever 7. Nausea and vomiting.

Have you experienced any complications following a urinary tract infection? 1. No 2. Yes

UTI antibiotics adherence (those who completed an antibiotic course as per the physician prescription without missing a dose are considered as "yes) 1.Yes  2. No

Any risk factors for UTI: a. Foley's catheter insertion in the past (within 6 months): Yes/No

b. Renal calculi: Yes/No c.Stricture Urethra: Yes/No

d.Benign prostatic hypertrophy (BPH): Yes/No

e.Cervicitis: Yes/No

Adherence to diabetes blood sugar monitoring: 1. No 2. Yes

Fasting blood sugar: 1. 80 -130 mg/dl 2.130 -200 mg/dl  3.>200 mg/dl 4. No idea 

HbA1C level % : …………
